# Supplementary material for: Project OPUS: Development and evaluation of an electronic platform for pain management education of medical undergraduates in resource-limited settings
Source: PLoS One. 2020 Dec 10;15(12):e0243573. doi: 10.1371/journal.pone.0243573 (PMC7728241; doi:10.1371/journal.pone.0243573)
Supplement: S1 File — Pain knowledge scores attained by students at pre-test, following completion of modules and at post-test. (PDF) [file pone.0243573.s001.pdf]

University site	ParticipantID	sex	pretest	posttest	universitymeanpretest	universitymeanposttest	Pretest	Mod1	Mod2	Mod3	Mod4	Mod5	Mod6	Posttest
1	1	0	0.40	0.65	0.37	0.59	0.40	0.80	1.00	0.80	0.80	0.80	1.00	0.65
1	2	0	0.35	0.50	0.37	0.59	0.35	0.80	0.60	0.20	0.80	0.80	0.80	0.50
1	3	0	0.40	0.60	0.37	0.59	0.40	0.80	0.80	0.60	0.80	0.60	0.80	0.60
1	4	0	0.50	0.65	0.37	0.59	0.50	0.80	1.00	0.80	0.80	0.80	0.80	0.65
1	5	1	0.20	0.70	0.37	0.59	0.20	0.80	0.80	0.60	0.80	1.00	0.80	0.70
1	6	0	0.40	0.70	0.37	0.59	0.40	0.80	0.60	1.00	0.60	0.80	1.00	0.70
1	7	0	0.35	0.60	0.37	0.59	0.35	0.80	0.80	0.80	0.80	0.60	0.80	0.60
1	8	1	0.35	0.50	0.37	0.59	0.35	0.80	1.00	0.80	0.80	1.00	0.80	0.50
1	9	1	0.30	0.55	0.37	0.59	0.30	0.60	0.80	1.00	0.60	0.60	0.80	0.55
1	10	0	0.40	0.70	0.37	0.59	0.40	0.80	0.60	0.60	0.80	0.60	0.60	0.70
1	11	1	0.50	0.55	0.37	0.59	0.50	0.80	1.00	0.60	0.60	1.00	1.00	0.55
1	12	1	0.40	0.60	0.37	0.59	0.40	0.60	1.00	0.80	0.80	0.80	1.00	0.60
1	13	1	0.45	0.50	0.37	0.59	0.45	0.80	1.00	1.00	0.80	0.60	1.00	0.50
1	14	0	0.30	0.75	0.37	0.59	0.30	0.80	0.80	0.80	0.60	1.00	0.80	0.75
1	15	1	0.25	0.50	0.37	0.59	0.25	0.60	1.00	0.60	0.80	0.80	1.00	0.50
1	16	0	0.50	0.50	0.37	0.59	0.50	0.60	1.00	1.00	0.80	0.60	1.00	0.50
1	17	1	0.30	0.60	0.37	0.59	0.30	0.80	0.60	0.60	0.80	1.00	1.00	0.60
1	18	0	0.20	0.60	0.37	0.59	0.20	0.60	0.80	0.80	0.80	0.80	0.80	0.60
1	19	0	0.35	0.50	0.37	0.59	0.35	0.60	0.60	0.80	0.80	0.60	1.00	0.50
1	20	0	0.45	0.55	0.37	0.59	0.45	0.80	1.00	0.80	0.80	0.80	0.80	0.55
2	1	0	0.30	0.65	0.42	0.59	0.30	0.80	0.80	0.80	0.60	0.80	0.80	0.65
2	2	0	0.45	0.50	0.42	0.59	0.45	0.80	1.00	1.00	0.80	0.80	1.00	0.50
2	3	0	0.45	0.75	0.42	0.59	0.45	0.60	1.00	1.00	0.80	1.00	0.80	0.75
2	4	1	0.45	0.50	0.42	0.59	0.45	0.80	0.80	0.80	0.60	0.60	0.80	0.50
2	5	1	0.45	0.50	0.42	0.59	0.45	0.80	0.80	1.00	0.80	1.00	1.00	0.50
2	6	0	0.55	0.55	0.42	0.59	0.55	0.60	1.00	1.00	0.80	0.60	1.00	0.55
2	7	1	0.30	0.75	0.42	0.59	0.30	0.80	1.00	1.00	0.80	0.80	0.60	0.75
2	8	0	0.25	0.35	0.42	0.59	0.25	0.60	0.60	0.80	0.80	0.80	1.00	0.35
2	9	1	0.40	0.65	0.42	0.59	0.40	0.80	0.60	0.80	0.60	0.80	0.60	0.65
2	10	1	0.30	0.60	0.42	0.59	0.30	0.60	0.80	1.00	0.80	0.80	1.00	0.60
2	11	1	0.35	0.60	0.42	0.59	0.35	0.60	0.80	0.80	0.60	0.80	0.50	0.60
2	12	0	0.30	0.70	0.42	0.59	0.30	1.00	0.80	0.80	0.80	0.80	0.80	0.70
2	13	1	0.55	0.65	0.42	0.59	0.55	0.80	0.80	0.80	0.80	0.80	0.80	0.65
2	14	0	0.50	0.75	0.42	0.59	0.50	0.80	1.00	1.00	0.80	1.00	0.80	0.75
2	15	0	0.60	0.35	0.42	0.59	0.60	0.80	0.80	0.80	0.80	0.80	1.00	0.35
2	16	1	0.65	0.60	0.42	0.59	0.65	0.80	1.00	0.80	0.80	0.80	1.00	0.60
2	17	1	0.35	0.55	0.42	0.59	0.35	0.60	1.00	1.00	0.80	0.80	1.00	0.55
2	18	1	0.50	0.85	0.42	0.59	0.50	0.80	0.80	1.00	0.80	0.80	0.80	0.85
2	19	0	0.50	0.60	0.42	0.59	0.50	0.80	1.00	0.80	0.80	0.80	1.00	0.60
2	20	0	0.50	0.60	0.42	0.59	0.50	0.80	1.00	1.00	0.80	0.80	0.80	0.60
2	21	0	0.30	0.75	0.42	0.59	0.30	0.60	1.00	1.00	0.80	0.80	1.00	0.75
2	22	0	0.45	0.35	0.42	0.59	0.45	0.80	1.00	0.80	0.80	0.80	1.00	0.35
2	23	0	0.30	0.55	0.42	0.59	0.30	0.80	0.80	0.80	1.00	0.80	0.80	0.55
2	24	0	0.45	0.75	0.42	0.59	0.45	0.80	0.80	0.80	0.80	0.80	1.00	0.75
2	25	1	0.60	0.80	0.42	0.59	0.60	0.80	1.00	1.00	0.80	0.80	0.80	0.80
2	26	0	0.40	0.55	0.42	0.59	0.40	0.60	0.80	1.00	0.80	0.80	0.80	0.55
2	27	1	0.60	0.70	0.42	0.59	0.60	0.60	0.60	0.80	0.60	0.80	1.00	0.70

2	28	1	0.50	0.50	0.42	0.59	0.50	0.80	0.80	0.60	0.80	0.80	0.80	0.50
2	29	0	0.55	0.70	0.42	0.59	0.55	1.00	1.00	0.80	0.80	1.00	0.80	0.70
2	30	1	0.40	0.30	0.42	0.59	0.40	0.80	0.80	0.80	0.80	0.80	0.80	0.30
2	31	0	0.40	0.55	0.42	0.59	0.40	0.60	1.00	1.00	0.80	0.80	1.00	0.55
2	32	1	0.55	0.65	0.42	0.59	0.55	0.80	1.00	1.00	0.80	0.80	1.00	0.65
2	33	1	0.45	0.60	0.42	0.59	0.45	0.60	0.80	0.80	0.60	0.80	1.00	0.60
2	34	1	0.45	0.90	0.42	0.59	0.45	0.60	0.80	0.80	0.80	0.80	1.00	0.90
2	35	1	0.25	0.75	0.42	0.59	0.25	0.80	0.80	0.60	1.00	1.00	0.80	0.75
2	36	1	0.25	0.55	0.42	0.59	0.25	0.75	0.80	1.00	0.60	0.80	1.00	0.55
2	37	1	0.60	0.50	0.42	0.59	0.60	0.80	1.00	1.00	0.80	0.80	1.00	0.50
2	38	1	0.30	0.80	0.42	0.59	0.30	0.80	0.80	0.80	0.80	1.00	1.00	0.80
2	39	0	0.40	0.55	0.42	0.59	0.40	0.80	1.00	0.80	0.80	0.80	0.80	0.55
2	40	0	0.40	0.40	0.42	0.59	0.40	0.40	0.80	0.80	0.80	0.80	0.80	0.40
2	41	0	0.35	0.65	0.42	0.59	0.35	0.80	1.00	0.60	0.80	1.00	1.00	0.65
2	42	0	0.60	0.50	0.42	0.59	0.60	0.80	1.00	0.80	0.80	0.80	1.00	0.50
2	43	0	0.30	0.55	0.42	0.59	0.30	0.80	1.00	0.80	0.80	1.00	1.00	0.55
2	44	1	0.40	0.75	0.42	0.59	0.40	0.80	1.00	1.00	0.80	0.80	0.80	0.75
2	45	1	0.30	0.60	0.42	0.59	0.30	0.80	0.80	0.80	0.80	0.60	0.80	0.60
2	46	1	0.60	0.30	0.42	0.59	0.60	0.60	0.60	0.60	0.80	0.80	0.80	0.30
2	47	0	0.35	0.45	0.42	0.59	0.35	0.60	0.80	0.80	0.60	0.80	1.00	0.45
2	48	1	0.50	0.60	0.42	0.59	0.50	0.80	0.80	1.00	0.80	1.00	0.80	0.60
2	49	1	0.45	0.75	0.42	0.59	0.45	0.80	0.80	1.00	0.80	1.00	1.00	0.75
2	50	0	0.35	0.65	0.42	0.59	0.35	0.80	1.00	1.00	0.80	0.80	0.60	0.65
2	51	1	0.30	0.55	0.42	0.59	0.30	0.80	0.60	1.00	0.60	0.80	1.00	0.55
2	52	0	0.45	0.45	0.42	0.59	0.45	0.45	0.80	0.80	1.00	0.80	0.60	0.45
2	53	0	0.55	0.60	0.42	0.59	0.55	0.80	1.00	1.00	0.80	0.60	1.00	0.60
2	54	1	0.60	0.60	0.42	0.59	0.60	0.80	1.00	0.80	0.80	1.00	1.00	0.60
2	55	1	0.30	0.60	0.42	0.59	0.30	0.80	0.80	0.80	0.80	0.80	0.80	0.60
2	56	1	0.60	0.65	0.42	0.59	0.60	0.80	1.00	0.80	0.80	0.80	1.00	0.65
2	57	1	0.40	0.75	0.42	0.59	0.40	0.80	0.60	0.80	0.80	1.00	0.80	0.75
2	58	0	0.45	0.65	0.42	0.59	0.45	0.60	0.60	0.80	0.60	0.80	0.80	0.65
2	59	1	0.50	0.35	0.42	0.59	0.50	0.80	0.80	0.80	0.80	0.80	0.80	0.35
2	60	0	0.35	0.45	0.42	0.59	0.35	0.80	1.00	0.80	0.80	0.80	0.80	0.45
2	61	1	0.35	0.45	0.42	0.59	0.35	0.80	0.80	0.80	0.80	0.80	0.40	0.45
2	62	1	0.40	0.60	0.42	0.59	0.40	0.80	0.80	0.80	0.80	0.80	0.80	0.60
2	63	0	0.50	0.45	0.42	0.59	0.50	0.80	1.00	1.00	1.00	1.00	1.00	0.45
2	64	1	0.50	0.60	0.42	0.59	0.50	0.80	1.00	1.00	0.80	1.00	1.00	0.60
2	65	1	0.40	0.55	0.42	0.59	0.40	0.40	1.00	1.00	1.00	0.60	0.80	0.55
2	66	1	0.60	0.60	0.42	0.59	0.60	0.60	1.00	1.00	0.60	0.80	0.60	0.60
2	67	0	0.35	0.70	0.42	0.59	0.35	0.80	0.80	0.60	0.80	0.60	1.00	0.70
2	68	0	0.30	0.45	0.42	0.59	0.30	0.80	1.00	1.00	0.80	0.80	0.80	0.45
2	69	1	0.50	0.80	0.42	0.59	0.50	0.80	0.80	1.00	0.80	1.00	1.00	0.80
2	70	0	0.30	0.50	0.42	0.59	0.30	0.80	0.60	0.60	0.80	0.80	1.00	0.50
2	71	0	0.30	0.55	0.42	0.59	0.30	0.80	1.00	0.80	0.80	0.60	1.00	0.55
2	72	1	0.35	0.45	0.42	0.59	0.35	0.80	1.00	0.80	0.80	0.80	1.00	0.45
2	73	1	0.40	0.45	0.42	0.59	0.40	0.80	1.00	0.80	0.80	0.80	1.00	0.45
2	74	0	0.55	0.60	0.42	0.59	0.55	0.60	0.60	0.60	0.80	0.60	0.60	0.60
2	75	0	0.50	0.50	0.42	0.59	0.50	0.80	0.80	1.00	0.80	1.00	1.00	0.50

2	76	0	0.45	0.60	0.42	0.59	0.45	0.80	0.60	0.80	0.60	0.60	0.60	0.60
2	77	0	0.60	0.75	0.42	0.59	0.60	0.60	0.80	1.00	0.80	1.00	1.00	0.75
2	78	1	0.70	0.75	0.42	0.59	0.70	0.80	1.00	0.80	0.80	0.80	0.80	0.75
2	79	0	0.45	0.55	0.42	0.59	0.45	0.80	0.60	1.00	0.80	1.00	1.00	0.55
2	80	1	0.20	0.55	0.42	0.59	0.20	0.80	0.80	1.00	0.80	0.80	0.80	0.55
2	81	0	0.40	0.60	0.42	0.59	0.40	0.80	0.80	0.80	0.80	0.80	0.80	0.60
2	82	1	0.30	0.50	0.42	0.59	0.30	0.60	0.80	0.60	0.80	0.80	0.60	0.50
2	83	1	0.35	0.50	0.42	0.59	0.35	0.80	0.80	0.80	0.80	0.80	1.00	0.50
2	84	0	0.65	0.75	0.42	0.59	0.65	0.80	0.80	0.80	0.80	0.80	0.80	0.75
2	85	1	0.25	0.65	0.42	0.59	0.25	0.80	0.80	0.80	0.80	0.80	1.00	0.65
2	86	0	0.30	0.65	0.42	0.59	0.30	0.80	0.80	0.80	0.80	0.80	0.80	0.65
2	87	0	0.30	0.70	0.42	0.59	0.30	0.60	1.00	1.00	0.80	0.60	0.77	0.70
2	88	1	0.45	0.55	0.42	0.59	0.45	0.40	0.80	1.00	0.60	0.80	0.80	0.55
2	89	1	0.25	0.45	0.42	0.59	0.25	0.80	0.80	1.00	0.80	0.80	0.80	0.45
2	90	1	0.30	0.50	0.42	0.59	0.30	0.80	0.80	1.00	0.80	0.80	1.00	0.50
2	91	0	0.25	0.50	0.42	0.59	0.25	0.80	1.00	0.80	0.80	0.80	0.80	0.50
2	92	0	0.25	0.65	0.42	0.59	0.25	1.00	1.00	0.80	0.80	0.80	1.00	0.65
2	93	0	0.25	0.60	0.42	0.59	0.25	0.80	0.80	0.80	0.80	1.00	1.00	0.60
3	1	0	0.25	0.50	0.44	0.64	0.25	0.60	1.00	1.00	0.80	0.80	1.00	0.50
3	2	0	0.35	0.85	0.44	0.64	0.35	1.00	1.00	0.80	0.80	1.00	1.00	0.85
3	3	0	0.50	0.50	0.44	0.64	0.50	0.60	1.00	0.80	0.80	0.80	1.00	0.50
3	4	0	0.50	0.60	0.44	0.64	0.50	0.60	1.00	0.80	0.80	0.60	1.00	0.60
3	5	0	0.70	0.80	0.44	0.64	0.70	0.60	1.00	1.00	0.80	1.00	1.00	0.80
3	6	1	0.50	0.85	0.44	0.64	0.50	0.60	0.80	0.60	0.80	1.00	1.00	0.85
3	7	0	0.50	0.45	0.44	0.64	0.50	0.60	0.80	0.60	0.80	0.60	0.60	0.45
3	8	0	0.40	0.65	0.44	0.64	0.40	0.60	0.80	1.00	0.80	1.00	1.00	0.65
3	9	1	0.35	0.60	0.44	0.64	0.35	0.60	1.00	0.80	0.80	0.80	1.00	0.60
3	10	1	0.35	0.55	0.44	0.64	0.35	0.40	0.80	0.80	0.80	0.80	0.80	0.55
4	1	0	0.35	0.90	0.41	0.69	0.35	0.80	1.00	1.00	0.60	1.00	1.00	0.90
4	2	0	0.40	0.50	0.41	0.69	0.40	0.60	0.60	0.80	0.80	0.80	0.60	0.50
4	3	0	0.50	0.65	0.41	0.69	0.50	0.80	0.80	0.60	0.60	0.60	0.60	0.65
4	4	0	0.30	0.50	0.41	0.69	0.30	0.60	0.60	0.60	0.80	0.80	0.60	0.50
4	5	0	0.35	0.55	0.41	0.69	0.35	0.80	0.60	0.80	0.60	0.60	1.00	0.55
4	6	0	0.55	0.60	0.41	0.69	0.55	0.60	0.80	0.60	0.60	0.60	0.60	0.60
4	7	0	0.45	0.70	0.41	0.69	0.45	0.80	0.60	1.00	0.60	0.60	0.60	0.70
4	8	0	0.30	0.65	0.41	0.69	0.30	0.80	0.80	0.80	0.80	0.80	1.00	0.65
4	9	0	0.85	0.55	0.41	0.69	0.85	0.80	1.00	1.00	0.80	1.00	1.00	0.55
4	10	0	0.40	0.50	0.41	0.69	0.40	0.60	0.80	0.60	0.60	0.80	0.60	0.50
4	11	0	0.35	0.60	0.41	0.69	0.35	0.80	0.60	0.80	0.80	1.00	1.00	0.60
4	12	0	0.45	0.55	0.41	0.69	0.45	0.80	0.80	1.00	0.80	0.80	0.80	0.55
4	13	0	0.40	0.95	0.41	0.69	0.40	0.80	1.00	0.80	0.80	1.00	0.80	0.95
4	14	1	0.45	0.55	0.41	0.69	0.45	0.80	0.60	0.80	0.60	0.80	0.60	0.55
4	15	1	0.55	0.55	0.41	0.69	0.55	0.80	1.00	1.00	0.80	0.80	1.00	0.55
4	16	1	0.55	1.00	0.41	0.69	0.55	0.80	1.00	1.00	0.80	1.00	1.00	1.00
4	17	1	0.35	0.85	0.41	0.69	0.35	0.60	0.60	0.80	0.60	1.00	0.80	0.85
4	18	0	0.35	0.55	0.41	0.69	0.35	0.80	1.00	1.00	0.80	1.00	1.00	0.55
4	19	1	0.30	1.00	0.41	0.69	0.30	0.80	1.00	1.00	0.80	1.00	1.00	1.00
4	20	1	0.45	0.60	0.41	0.69	0.45	0.80	0.80	0.80	0.80	0.80	0.60	0.60

4	21	0	0.30	0.95	0.41	0.69	0.30	0.60	1.00	1.00	0.80	1.00	1.00	0.95
4	22	0	0.40	0.55	0.41	0.69	0.40	0.60	0.60	0.60	0.60	0.60	0.60	0.55
4	23	1	0.30	0.65	0.41	0.69	0.30	0.80	0.60	0.60	0.80	1.00	0.80	0.65
4	24	1	0.30	0.95	0.41	0.69	0.30	1.00	0.20	1.00	0.80	1.00	1.00	0.95
4	25	1	0.30	0.95	0.41	0.69	0.30	0.80	1.00	1.00	0.80	1.00	1.00	0.95
4	26	0	0.30	0.75	0.41	0.69	0.30	0.80	0.80	0.60	0.80	0.60	1.00	0.75
4	27	1	0.25	0.90	0.41	0.69	0.25	0.60	0.60	0.60	0.80	0.80	1.00	0.90
4	28	1	0.35	0.60	0.41	0.69	0.35	0.80	0.60	0.60	0.60	0.60	0.60	0.60
4	29	0	0.45	0.55	0.41	0.69	0.45	0.60	0.80	0.80	0.80	0.80	1.00	0.55
4	30	1	0.45	0.30	0.41	0.69	0.45	0.80	0.80	0.80	0.80	0.80	0.80	0.30
4	31	1	0.40	0.65	0.41	0.69	0.40	0.80	1.00	1.00	0.80	1.00	1.00	0.65
4	32	1	0.60	0.70	0.41	0.69	0.60	0.60	1.00	1.00	0.80	1.00	1.00	0.70
4	33	1	0.20	0.50	0.41	0.69	0.20	0.60	0.80	0.80	0.80	0.80	1.00	0.50
4	34	1	0.35	0.95	0.41	0.69	0.35	1.00	1.00	1.00	0.80	1.00	0.80	0.95
4	35	1	0.25	1.00	0.41	0.69	0.25	0.80	1.00	1.00	0.80	1.00	1.00	1.00
4	36	0	0.75	0.95	0.41	0.69	0.75	0.80	1.00	1.00	0.80	1.00	1.00	0.95
4	37	1	0.45	0.25	0.41	0.69	0.45	0.80	1.00	1.00	0.80	0.60	1.00	0.25
4	38	0	1.00	0.95	0.41	0.69	1.00	0.80	1.00	1.00	0.80	1.00	1.00	0.95
4	39	1	0.35	0.80	0.41	0.69	0.35	0.60	0.60	0.60	0.60	1.00	1.00	0.80
4	40	1	0.35	0.60	0.41	0.69	0.35	0.60	0.60	1.00	0.60	0.80	0.60	0.60
4	41	0	0.35	0.75	0.41	0.69	0.35	0.60	1.00	1.00	0.80	1.00	0.80	0.75
4	42	0	0.30	0.60	0.41	0.69	0.30	0.80	1.00	1.00	0.60	0.60	0.80	0.60
4	43	1	0.45	0.95	0.41	0.69	0.45	0.60	0.80	0.80	0.80	1.00	0.80	0.95
4	44	1	0.30	0.25	0.41	0.69	0.30	0.80	1.00	0.80	0.80	0.60	1.00	0.25
4	45	0	0.35	0.55	0.41	0.69	0.35	0.80	0.80	0.80	0.80	0.60	1.00	0.55
4	46	1	0.40	0.40	0.41	0.69	0.40	0.80	0.40	0.40	0.80	0.40	0.20	0.40
4	47	1	0.25	0.60	0.41	0.69	0.25	0.60	0.60	0.80	0.80	0.80	0.80	0.60
4	48	1	0.40	0.50	0.41	0.69	0.40	0.80	0.80	1.00	0.80	1.00	1.00	0.50
4	49	1	0.30	1.00	0.41	0.69	0.30	0.80	1.00	1.00	1.00	1.00	1.00	1.00
4	50	1	0.25	0.55	0.41	0.69	0.25	0.60	0.60	0.80	0.80	0.80	0.80	0.55
4	51	0	0.45	0.55	0.41	0.69	0.45	0.80	1.00	1.00	0.80	0.80	1.00	0.55
4	52	1	0.65	0.65	0.41	0.69	0.65	0.80	1.00	0.80	0.80	0.80	1.00	0.65
4	53	1	0.30	0.95	0.41	0.69	0.30	0.80	1.00	1.00	0.80	1.00	1.00	0.95
4	54	1	0.45	0.40	0.41	0.69	0.45	0.80	0.80	1.00	0.80	0.80	1.00	0.40
4	55	1	0.45	0.85	0.41	0.69	0.45	0.40	0.60	0.60	0.80	0.80	0.40	0.85
4	56	0	0.60	0.50	0.41	0.69	0.60	0.80	0.60	0.60	0.60	0.60	0.60	0.50
4	57	0	0.40	0.50	0.41	0.69	0.40	0.60	0.60	0.60	0.80	1.00	1.00	0.50
4	58	0	1.00	0.95	0.41	0.69	1.00	0.80	1.00	1.00	0.80	1.00	0.60	0.95
4	59	0	0.40	0.50	0.41	0.69	0.40	0.80	0.80	1.00	0.80	0.60	1.00	0.50
4	60	0	0.30	0.50	0.41	0.69	0.30	0.60	0.60	0.60	0.60	0.80	0.60	0.50
4	61	1	0.30	0.95	0.41	0.69	0.30	0.80	0.80	1.00	0.60	0.60	0.80	0.95
4	62	1	0.25	0.95	0.41	0.69	0.25	0.80	1.00	1.00	0.80	1.00	1.00	0.95
4	63	0	0.35	0.85	0.41	0.69	0.35	0.80	1.00	1.00	0.80	1.00	1.00	0.85
4	64	0	0.35	0.65	0.41	0.69	0.35	0.80	1.00	0.80	0.80	1.00	1.00	0.65
4	65	0	0.25	0.50	0.41	0.69	0.25	0.40	0.80	0.80	0.80	0.80	0.80	0.50
4	66	0	0.35	1.00	0.41	0.69	0.35	0.80	1.00	1.00	0.80	1.00	1.00	1.00
4	67	1	0.40	0.60	0.41	0.69	0.40	0.80	0.60	1.00	0.80	0.80	0.60	0.60
4	68	1	0.35	1.00	0.41	0.69	0.35	1.00	1.00	1.00	0.80	1.00	1.00	1.00

4	69	0	0.45	0.60	0.41	0.69	0.45	0.60	1.00	1.00	0.80	1.00	1.00	0.60
4	70	1	0.50	0.55	0.41	0.69	0.50	0.60	0.60	0.60	0.60	0.60	0.80	0.55
4	71	1	0.30	0.25	0.41	0.69	0.30	0.80	1.00	0.80	1.00	1.00	1.00	0.25
4	72	1	0.55	0.95	0.41	0.69	0.55	0.80	1.00	1.00	0.80	1.00	1.00	0.95
4	73	0	0.35	0.90	0.41	0.69	0.35	0.60	1.00	1.00	0.80	1.00	1.00	0.90
4	74	1	0.40	0.85	0.41	0.69	0.40	0.80	1.00	1.00	0.80	1.00	1.00	0.85
4	75	1	0.55	0.65	0.41	0.69	0.55	0.60	1.00	1.00	0.80	1.00	1.00	0.65
4	76	1	0.30	0.85	0.41	0.69	0.30	0.80	1.00	1.00	0.80	1.00	0.80	0.85
4	77	0	0.95	0.95	0.41	0.69	0.95	0.80	1.00	1.00	0.80	1.00	1.00	0.95
4	78	1	0.25	0.50	0.41	0.69	0.25	0.60	0.80	0.60	0.80	0.60	1.00	0.50
4	79	0	0.30	0.65	0.41	0.69	0.30	0.60	0.60	0.80	0.80	0.60	0.80	0.65
4	80	0	0.50	0.50	0.41	0.69	0.50	0.60	1.00	0.60	0.80	0.60	1.00	0.50
4	81	1	0.40	0.50	0.41	0.69	0.40	0.60	1.00	1.00	0.80	1.00	1.00	0.50
4	82	0	0.55	0.65	0.41	0.69	0.55	0.60	0.80	1.00	0.80	0.60	1.00	0.65
4	83	0	0.30	0.95	0.41	0.69	0.30	0.60	1.00	0.60	0.60	1.00	1.00	0.95
4	84	0	0.35	0.65	0.41	0.69	0.35	0.60	1.00	1.00	0.80	1.00	1.00	0.65
4	85	0	0.40	0.65	0.41	0.69	0.40	0.60	0.80	0.80	0.80	0.80	0.80	0.65
4	86	1	0.25	0.50	0.41	0.69	0.25	0.60	0.60	1.00	0.60	0.80	1.00	0.50
4	87	1	0.20	1.00	0.41	0.69	0.20	0.60	1.00	1.00	0.60	1.00	1.00	1.00
4	88	1	0.35	0.60	0.41	0.69	0.35	0.60	0.60	1.00	0.80	1.00	0.80	0.60
5	1	0	0.30	0.40	0.38	0.60	0.30	0.80	1.00	1.00	0.80	0.80	1.00	0.40
5	2	0	0.40	0.75	0.38	0.60	0.40	0.80	1.00	1.00	0.80	0.80	1.00	0.75
5	3	0	0.55	0.60	0.38	0.60	0.55	0.60	1.00	1.00	0.80	0.80	0.80	0.60
5	4	0	0.50	0.50	0.38	0.60	0.50	0.80	0.80	0.80	0.80	0.80	1.00	0.50
5	5	1	0.30	0.65	0.38	0.60	0.30	0.60	1.00	1.00	0.80	0.80	1.00	0.65
5	6	1	0.40	0.60	0.38	0.60	0.40	0.60	0.80	1.00	0.80	0.60	1.00	0.60
5	7	1	0.30	0.75	0.38	0.60	0.30	0.60	1.00	0.80	0.80	0.80	1.00	0.75
5	8	0	0.30	0.55	0.38	0.60	0.30	0.80	0.80	0.60	0.80	0.80	1.00	0.55
