# Supplementary material for: Project OPUS: Development and evaluation of an electronic platform for pain management education of medical undergraduates in resource-limited settings
Source: PLoS One. 2020 Dec 10;15(12):e0243573. doi: 10.1371/journal.pone.0243573 (PMC7728241; doi:10.1371/journal.pone.0243573)
Supplement: Attachment — Submitted filename: Response to Reviewers.doc [file pone.0243573.s002.doc]

Thank you for the positive feedback received from reviewers 1 and 2, and the editorial team, and for the opportunity to respond to the constructive points in our accompanying revised manuscript. Please find below our point-by-point response to reviewer feedback outlining, where relevant, the related changes we have made.

| **Reviewer comments** | **Author response** |
| --- | --- |
| ***Editorial feedback*** | |
| Mention in the limitations of the study that only knowledge acquisition was examined without considering skills and attitude   - Reviewer 1: Knowledge acquisition – what about skills and attitude? Should be mentioned as a limitation. - Reviewer 2: A strong discussion. Some acknowledgement of the limitations around the smaller sub-groups (sites) and statistical analysis would be important. | Many thanks for these important points. Both have been incorporated into the limitations section of the manuscript. |
| Show is there is any feedback about comparison of App with other platforms   - Reviewer 1: App vs. other platforms? Any feedback? | We did not obtain specific data comparing the app against other platforms from study participants. However, in the findings we do outline findings from benchmarking of the app against other online tools using the UEQ Data Analysis Tool, indicating that the e-Learning platform was rated above average and good across the majority of domains it covers. |
| Clarify if there is any Interactive element in the e-learning system   - Reviewer 1: Was there any Interactive element? To what extent? | The e-Learning system contained limited interactive elements, such as the selection of the courses by students and options to view or skip any course, alongside the ability to take and retake the tests. However, the content was presented as static written content and videos. Students could access and view this material, but there were no interactive elements to the content itself. Our focus on development and evaluation was, in part, to determine the feasibility of the approach. We have included detail about this in the manuscript. Future plans for the platform include developing more interactive content. |
| Clarify if the e-learning system can be used only for self-learning or student interaction could be allowed with the teacher and/or with the other students | The delivery of eLearning platform was designed for self-learning. There is now a student and teacher interaction function within the platform, enabling messages to be shared. However, this function was under development during the Project OPUS project. |
| Use latest IASP 2020 pain definition   - Reviewer 1: Pain definition has been revised recently – use latest IASP definition 2020 - Reviewer 2: IASP definition of pain has recently been updated and needs to be reflected in the paper | The revised IASP 2020 pain definition has been inserted in the manuscript and the IASP 1979 pain definition has been removed. |
| Mention other similar courses on pain in developed countries – UK, Europe, Australia, Canada   - Reviewer 1: Please mention other similar courses on pain in developed countries – UK, Europe, Australia, Canada | We have included a citation to similar courses that have been developed across high-income settings, listing specific countries, in the introduction section. |
| Discuss implications that VTR Mobile is a proprietary item – or mention under limitations   - VTR Mobile – proprietary item – concerns about dependence on this particular proprietary item? Needs discussion or mention under limitations. | We have added content to the discussion section which addresses this important point. Part of the reason we selected the technology partner was because of their track record of working with the government in Nigeria, but we do need to consider how access to the content can be achieved without cost to students and will seek to align our future development plans with emerging frameworks aiming to enhance the effectiveness of e-learning as an educational tool to increase the quantity and quality of medical education programs. |
| Correct the citation style per journal requirement   - Reviewer 1: Please correct the citation style per Journal requirement (number of authors before et al. can be used). | We have amended the citation style per the journal requirements. |
| Discuss with more detail differences between completers and non-completers   - Reviewer 1: Only 1/3rd of the eligible students completed the course – difference between completers and non-completers? Selection bias potential. Has been briefly discussed under limitations, but a comparison would help. | We acknowledge the potential for selection bias in the recruitment of participants. We agree that it would be a helpful addition to compare completers with non-completers. However, demographic data was captured as part of the eLearning platform so only provided by those who engaged with the platform. Furthermore, our ethical approval did not include consent to access demographic data on non-completers from site records. In subsequent development and evaluation of the platform we will be keen to explore options for determining the sociodemographic characteristics of the target population to enable such a comparison. |
| Write more concisely some areas and summarise the development in a flow diagram   - Reviewer 2: Some areas of the paper could be more concisely written which is difficult with a complex project and data. - Reviewer 2: Methods: This paper describes the detailed development of the platform and curriculum which is one of its strengths. Anyone developing something similar would find this information helpful. However, some areas could be more concisely written and the development could be summarised in a flow diagram to reduce the word count. | We have reviewed the manuscript and made edits where we felt the writing could be more precise, but we agree that it is difficult to reduce further given the level of detail we have provided to support reproducibility of our approach and presenting the data collected. We felt that reducing the word count slightly to enable a more succinct description would be preferable to losing important detail and transparency through reducing the content into a flow diagram. |
| Specify guiding pedagogical theories of online/e-learning that supported the development of the curriculum   - Reviewer 1: Was it at appropriate stage for the students to grasp? What is the medical education system there? It would help the international reader. - Reviewer 1: Any reason why pass threshold was set at 70% (Table 1)? Page 13 last sentence says 80%. Please clarify. - Reviewer 2: As well as the review to determine the pain-related content, were there any guiding pedagogical theories of online/e-learning that supported the development of the curriculum? | Most of the project team are experienced teaching staff responsible for the delivery of training to medical undergraduates in the regions of Nigeria included in the study. The eLearning platform was developed at an appropriate level for the 5th year medical students who were the intended users. The 5th year is considered the midway point of the clinical years of medical school in Nigeria. In the 4th year, which is the beginning of the clinical training, students are introduced to medicine and surgery. By the 5th year, the curriculum includes paediatrics, obstetrics and gynaecology as well as community medicine. This is followed by clinical training in medicine and surgery in the 6th and final year of study. This context and positioning of the platform content have been reflected in the manuscript.  The pass threshold of 80% was set by consensus across the project team, determined as a necessary level of knowledge required prior to progression to the next module. The mention of 70% within Table 1 is an error and has been corrected.  We utilised the e-learning systems’ theoretical framework to guide the design of the platform, comprising three components: people, technologies, and services. The feasibility, practicalities and relevance of the different components were discussed. The ‘people’ component was covered through wider discussion and consultation across the research team, technology developers and university sites. This consultation guided both the technologies and services components of the platform. We determined through consultation that it would not be possible to incorporate the online platform into the existing undergraduate curriculum. The platform would have to supplement existing teaching activities and provide a standalone resource for students to access with appropriate technologies and services components. Consequently, an open learning model was adopted as a pedagogical framework, enabling flexibility and inclusivity of access to the platform content. A combination of instructional strategies was incorporated into the presentation of content, including contextualising instruction, activating and assessing learner outcomes and presenting and cueing content.  The above points in response to the three reviewer comments have been incorporated into the revised manuscript. |
| ***Additional amendment requests*** | |
| Reviewer 1: Table 5: cite only the median with IQR data, not the mean and SD data. | The mean and SD data have been removed from Table 5. |
| Reviewer 2: Introduction: Please consider revising this as representing pain as a complaint suggests that it is minor, a symptom rather than a complex condition or experience that can have a major impact on people. Also, this emphasizes the reporting of pain and there are many who cannot express pain or are reluctant to do so. | Thank you for raising this. We have reworded the opening statement to describe pain as a symptom that is commonly reported by patients presenting to health professionals. We feel the revised wording captures the wide prevalence of pain being reported without misrepresenting the significance of its impact of those experiencing it. |
| Reviewer 2: Introduction: Overview of platform development – HIT acronym needs explaining/writing out | Apologies, this was an oversight on our part. There is only one instance of ‘health information technology’ mentioned. We have replaced the acronym with the term in full. |
| Reviewer 2: Methods: Research tools: Knowledge survey –what was covered in this? Pain Beliefs Survey – reference and details needed. Whether reliability and validity testing was conducted on these is needed | The development and testing of the survey have been reported and published elsewhere. We have included a citation of this work in the manuscript. |
| Reviewer 2: Methods: Page 15 - In this sentence, change done to assessed ‘The normality of distribution of the data was done…’ and later in the same paragraph – undertaken is another option | Thank you for these suggestions. Both have been incorporated into the manuscript. |
| Reviewer 2: Results: Page 16, third line- should this be completion rate rather than retention rate? And again two lines later. Not sure retention is the correct term | Thank you for this suggestion. We have changed retention to completion in both instances. |
| Reviewer 2: Results: Table 3 states that 8 people completed the pre and post test from the ESUTH site but in Figure 2, this number is 5. Please can this be corrected with whichever is correct. | This was an error in Figure 2. We have amended this and submitted a revised file for Figure 2. |
| Reviewer 2: Results: Table 7 presents the qualitative analysis but it is unclear why a table has been used to present nearly 900 words of text. Consider revising. | We were keen to include the findings from the qualitative analysis as these offer insights into the experience of users. As highlighted by the reviewers, this is a detailed overview of the development process that was followed by our team. We have tried to ensure the description and findings are comprehensive but concise. We felt that qualitative findings, presented in a table with key points summarised in the text, was the best way of presenting the data without lengthening the narrative around the system development. However, the qualitative data is available and tabulated for those who would like to review the detail of interviews. We are happy to revise if required, but would like to keep the qualitative data in its current format if possible. |
| Reviewer 2: Conclusion: First line needs revision as it raises much broader issues that are not the focus of this paper, particularly opioid abuse/misuse. The line reads ‘Inadequate pain education will perpetuate existing public health problems of undertreated, pain, untreated pain and opioid abuse | Thank you for raising this point. The first sentence has been removed. |
