# Supplementary material for: Project OPUS: Development and evaluation of an electronic platform for pain management education of medical undergraduates in resource-limited settings
Source: PLoS One. 2020 Dec 10;15(12):e0243573. doi: 10.1371/journal.pone.0243573 (PMC7728241; doi:10.1371/journal.pone.0243573)
Supplement: Attachment — Submitted filename: Response to Reviewers.doc [file pone.0243573.s003.doc]

Thank you for the positive feedback and constructive points to improve our manuscript. Please find below our point-by-point response to reviewer feedback outlining, where relevant, the related changes we have made.

| **Reviewer comments** | **Author response** |
| --- | --- |
| ***Editorial feedback*** |  |
| It is recommended to carefully revise the manuscript including modifications suggested by reviewer 3, correcting typos (e. g. in section data analysis "pre-and post-test", page 8 "pre and post-test", "user-centered design[25]", "retirement.[24]") and eliminating discrepancies in notation (e .g. page 18 "Pre and post test scores " and "The pre-test, post-test...", Table 5 "Median Posttest"). | Many thanks for the opportunity to respond to your comments and those of the reviewers.  We have undertaken a close examination of the revised manuscript. We have corrected the typos and notation that you have highlighted and checked that no further issues remain. We outline our response to comments from reviewer 3 in more detail, below.  We have also processed also figure files using the PACE system and have attached the updated figure files to the submission. |
| ***Reviewer 1*** | |
| All the comments have been responded to my satisfaction. Corrections and changes have been made where possible and needed, and mentioned as limitations where needed but not possible. This version appears acceptable to me now. Congratulations on a very important work! | Many thanks for your comments and constructive guidance on how to develop the manuscript in previous iterations. |
| ***Reviewer 3*** | |
| This original research paper evaluated the implementation of a voluntarily 12-week e-learning course on pain education across five medical schools in Nigeria, with positive outcomes in regards to implementation of the method of e-learning and increased knowledge on pain. The advantages and limitations of this study have been nicely discussed. I want to congratulate the authors on their nice work on an important topic. | Thank you very much for your positive appraisal of our work. |
| 1. The whole reference list needs to be revised due to errors and mixups. As an example, see refs 28 and 33. | We have reviewed the reference list and ensured erroneous references have now been amended. Each reference has now been checked and verified. The referencing issues were due to an inadvertent issue with the formatting of references during the previous revision and has now been resolved. |
| 2. In the abstract, result section, perhaps it would be nice to state the collective pre- and post test scores across all medical schools in numbers and not only mention the good effect sizes. If word count is an issue I suggest removing (for instance) the mention of what statistical test used to compare pre- and post scores. | Many thanks for this suggestion. We have altered the text in the results section of the abstract to include a summary of increased knowledge. As suggested, to reduce words elsewhere we have made surrounding sections more concise. |
| 3. Methods. Very precisely described in regards to the platform. However, I would like some more information on how the selection of different sections from the IASP curriculum was made. Information on why the whole curriculum was not used and why additional resources were used to form the teaching material for this course. I think you should also add a reference to the specific curriculum outline used on the IASP website (if any). | We have included further detail in the overview of the construction and elaboration phase of development. In particular, on page 11, we have outlined how the IASP pain curriculum for medical graduates provided an initial structure to the e-Learning platform content, and was supplemented by context-specific information from the African Palliative Care Association publication, Beating Pain. We have also added further detail about the content that was shared during two iterations of development with support from an expert panel during the curriculum development. We have also included a citation of the IASP website where it is possible to access details of the IASP pain curriculum for medical graduates which was used to inform the structure and content of the e-Learning platform. |
| 4. On page 14, Method section; it is stated that the students were able to retake the tests. I recommend stating if this relates to the tests taken after completing each section (or if it also includes the pre/post tests.) | Thank you for highlighting the need for clarification. We have specified that repeating of tests was only possible for end of module tests, not the pre- and post-tests. This has been reflected in the manuscript on page 15. |
| 5. The pain beliefs survey mentioned in the methods; as far as I can see these results are not presented in this paper and the reference to its validation is not made on the students from this study, so why is this mentioned in the methods? Please clarify. | Data on pain beliefs was collected as a separate sub-study within this project using a pain beliefs scale developed by one of the co-authors (TO). We planned to present data from the pain beliefs survey alongside data from the e-Learning modules, but there was not a clear narrative and the data is instead being written up as a separate manuscript. We have removed any reference to the pain belief survey from the manuscript. |
